# The Tribulations of Trials: Lessons Learnt Recruiting 777 Older Adults Into REtirement in ACTion (REACT), a Trial of a Community, Group-Based Active Aging Intervention Targeting Mobility Disability

**DOI:** 10.1093/gerona/glaa051

**Published:** 2020-03-09

**Authors:** Janet Withall, Colin J Greaves, Janice L Thompson, Jolanthe L de Koning, Jessica C Bollen, Sarah J Moorlock, Kenneth R Fox, Max J Western, Tristan Snowsill, Antonieta Medina-Lara, Rosina Cross, Peter Ladlow, Gordon Taylor, Vasiliki Zisi, James Clynes, Selena Gray, Sandra Agyapong-Badu, Jack M Guralnik, W Jack Rejeski, Afroditi Stathi

**Affiliations:** 1 Department for Health, University of Bath, UK; 2 School of Sport, Exercise and Rehabilitation Sciences, University of Birmingham, UK; 3 University of Exeter Medical School, St Luke’s Campus, UK; 4 Centre for Exercise, Nutrition and Health Sciences, School for Policy Studies, University of Bristol, UK; 5 Institute of Health Research, College of Medicine and Health, South Cloisters, University of Exeter, St Luke’s Campus, UK; 6 Academic Department of Military Rehabilitation (ADMR), Defence Medical Rehabilitation Centre (DMRC) Stanford Hall, Loughborough, UK; 7 Department of Physical Education and Sport Sciences, University of Thessaly, Trikala, Greece; 8 Faculty of Health and Applied Sciences (HAS), University of the West of England (UWE Bristol), Frenchay Campus, Bristol, UK; 9 Department of Epidemiology and Public Health, University of Maryland, School of Medicine, Baltimore; 10 Department of Health and Exercise Science, Wake Forest University, Winston-Salem, North Carolina

**Keywords:** Physical activity, Physical function, Randomized control trial

## Abstract

**Background:**

Challenges of recruitment to randomized controlled trials (RCTs) and successful strategies to overcome them should be clearly reported to improve recruitment into future trials. REtirement in ACTion (REACT) is a United Kingdom-based multicenter RCT recruiting older adults at high risk of mobility disability to a 12-month group-based exercise and behavior maintenance program or to a minimal Healthy Aging control intervention.

**Methods:**

The recruitment target was 768 adults, aged 65 years and older scoring 4–9 on the Short Physical Performance Battery (SPPB). Recruitment methods include the following: (a) invitations mailed by general practitioners (GPs); (b) invitations distributed via third-sector organizations; and (c) public relations (PR) campaign. Yields, efficiency, and costs were calculated.

**Results:**

The study recruited 777 (33.9% men) community-dwelling, older adults (mean age 77.55 years (*SD* 6.79), mean SPPB score 7.37 (*SD* 1.56)), 95.11% white (*n* = 739) and broadly representative of UK quintiles of deprivation. Over a 20-month recruitment period, 25,559 invitations were issued. Eighty-eight percent of the participants were recruited via GP invitations, 5.4% via the PR campaign, 3% via word-of-mouth, and 2.5% via third-sector organizations. Mean recruitment cost per participant was £78.47, with an extra £26.54 per recruit paid to GPs to cover research costs.

**Conclusions:**

REACT successfully recruited to target. Response rates were lower than initially predicted and recruitment timescales required adjustment. Written invitations from GPs were the most efficient method for recruiting older adults at risk of mobility disability. Targeted efforts could achieve more ethnically diverse cohorts. All trials should be required to provide recruitment data to enable evidence-based planning of future trials.

## Background

Healthy aging is defined as “the process of developing and maintaining the functional ability that enables wellbeing in older age” (1). Functional ability is comprised the intrinsic capacities both mental and physical that people can draw on, relevant environmental characteristics and demands and how people respond to these demands. During old age, there is a population-wide transition from independence and adequate physical function toward frailty and an associated increased demand for health and social care support services (2–4). The impact of this trend is further heightened by an ever-increasing aging population in the United Kingdom (18.2% in 2017 over age 65 years rising to 20.7% by 2027) (5) that is reflected worldwide (6,7). Despite the positive impact of physical activity on slowing or preventing disability in later life (8,9), as people age, they engage in less physical activity and spend more time being sedentary (10,11). This toxic mix has increased the focus on methods to support the maintenance of functional capacity in later life, with healthy aging identified as a key public health priority (12,13).

The Retirement in Action (REACT) study was designed to assess the effectiveness and cost-effectiveness of a group-based exercise and socioeducational intervention for reducing the progression of functional limitations in older adults at high risk for mobility disability (14). Participants (*n* = 777) were randomized to either a 12-month exercise intervention or to a minimal control intervention.

Recruiting into large randomized controlled trials (RCTs) such as REACT is complex and challenging. A recent review reported that, between 2004 and 2016, *n* for recruitment target sample size was achieved in only 56% of 115 RCTs funded by the UK NIHR Health Technology Assessment program (15). Failing to reach recruitment targets within planned trial timescales and budgets can waste public funds. Despite the serious challenges of recruitment of representative samples and the value of information on recruitment for planning of future research, most studies report minimal information on their recruitment strategies, recruitment rates, relative yields and costs, and the lessons learned (15). However, through detailed and clear reporting of the recruitment processes, challenges, timescales, and required resources, those developing future studies can make realistic plans, avoid committing to overly optimistic timescales, and, through adoption of successful strategies, improve the cost-effectiveness of recruitment.

This article provides insights into the recruitment processes and baseline characteristics of the recruited sample of REACT participants. We describe the cost, strategies, and feasibility of recruiting at-risk community-dwelling older adults, as well as successes, challenges, and lessons learned. Based on these data, we provide guidance for researchers seeking to recruit older adults at risk for mobility disability into active aging trials, but which may also be useful for recruitment of older people to other types of activity promotion programs.

## Methods

### Trial Design

Designed to test real world delivery of a program informed by the successful Lifestyle Interventions and Independence for Elders Study (LIFE) in the United States (16,17), REACT is a multicenter, pragmatic, two-arm, parallel-group RCT with an internal pilot study incorporating comprehensive process and economic evaluations. The intervention comprised a standardized 12-month exercise program delivered in leisure/community centers by qualified exercise professionals with up to 15 participants per group. Sessions were twice a week for the first 12 weeks then once a week for the following 40 weeks. Each exercise-based training session incorporated components of strength, balance, and aerobic conditioning that were progressive and individually tailored to meet the participant’s functional needs. Sessions were followed by a short social session but from weeks 9 to 24, this was replaced by a weekly 45-minute interactive educational/social session run by the REACT trainers using evidence-based, person-centered behavior change strategies to build intrinsic motivation and self-efficacy. At 24 weeks, these educational sessions reduced to once a month. The control intervention comprised three 60–90-minute group sessions across the 2 years of the study consisting of presentations and discussion groups on aspects of healthy aging including topics such as healthy eating and dementia but excluding physical activity.

The study was reviewed and approved by the National Health Service (NHS), South East Coast-Surrey Research Ethics Committee (15/LO/2082). Outcome data were collected at baseline, 6, 12, and 24 months. Full details of the trial design are published elsewhere (14).

### Eligibility Criteria

Participants were community-dwelling adults, aged 65 years and older at risk of mobility disability but who were still ambulatory. This was assessed using the Short Physical Performance Battery (SPPB), including balance, gait speed, and timed sit-to-stand. The SPPB generates a physical function score from 0 to 12; older adults with scores of 4–9 inclusive were eligible to participate in the REACT study. Full eligibility criteria are reported elsewhere but key exclusion criteria included medical conditions that would preclude participation in gentle physical activity, living in residential or nursing care, and an inability to attend scheduled REACT PA sessions (14).

### Recruitment Strategies

Three trial sites (Bath/Bristol, Devon, and Birmingham) were chosen that represented urban, suburban, and semirural locations with diverse socioeconomic and ethnic characteristics. The main recruitment strategies utilized primary care (letters from general practitioners [GPs]; see Supplementary Appendix 1 for details), third-sector organizations (community groups, social enterprises, and sheltered housing facilities), word-of-mouth, and snowball techniques (friends, relations, or spouses of invitees). These approaches were supported by a low cost (£726) public relations (PR) campaign. A close working relationship was established with local community groups, charities, and the public sector, which facilitated events to present and discuss the study with their service users and issued written invitations to participate.

Based on the studies recruiting similar populations, we predicted that to achieve our target of 768 people, we would need to contact approximately 9,000 over a 14-month recruitment period (14,18,19). As the majority of these invitations would be issued by GPs, we estimated we needed to recruit 5–8 GP practices per site (15–24 in total), each with 9,000–13,000 patients (19). This was calculated based on the knowledge that 21.3% of people in Devon were aged 65 years and older, and an estimated prevalence of eligible people of 200 per thousand in the over-65 year population. The GPs involved in recruitment were not part of the study team but were recruited solely to run searches, identify eligible patients, and issue invitations to them.

During recruitment, participants were informed that they would receive shopping vouchers to the value of £15 (c$19) on attending each of the 6-, 12-, and 24-month assessment sessions.

### Screening and Randomization

Potential participants responded by returning a reply card, emailing or telephoning the research team. The response rate from the GP mailing was predicted to be 22% (*n* = 1,980) based on prior observational studies recruiting a similar population (20). After gaining verbal consent, those responding were telephone screened to check inclusion and exclusion criteria. We expected to exclude approximately 20% during this process. Eligible participants were then invited to a face-to-face screening and assessment session where written informed consent was obtained and participants could ask additional questions about the study. These sessions were held in local community centers and accessed via a range of transport methods: walking, public bus, car, or taxi. The SPPB was conducted and those scoring 4–9 were subsequently randomized. We anticipated this would be approximately 48% of those screened face-to-face. This would yield 8.5% of those originally approached who would meet the eligibility criteria (14) and a recruitment rate of 55 participants per month.

GP recruiters identified lists of patients who potentially met our inclusion criteria using computerized searches of their patient databases, which include field codes for age and a range of medical conditions. However, criteria such as employment status, current levels of physical activity, and physical function are not routinely captured on GP databases. GPs manually screened the computer-generated lists and excluded any patients for whom they deemed REACT would be inappropriate (uncontrolled heart condition, terminal condition, recent bereavement, etc.) (14). The database was then sent to a secure mailing house where invitations were printed, collated, and mailed.

**Figure 1.**
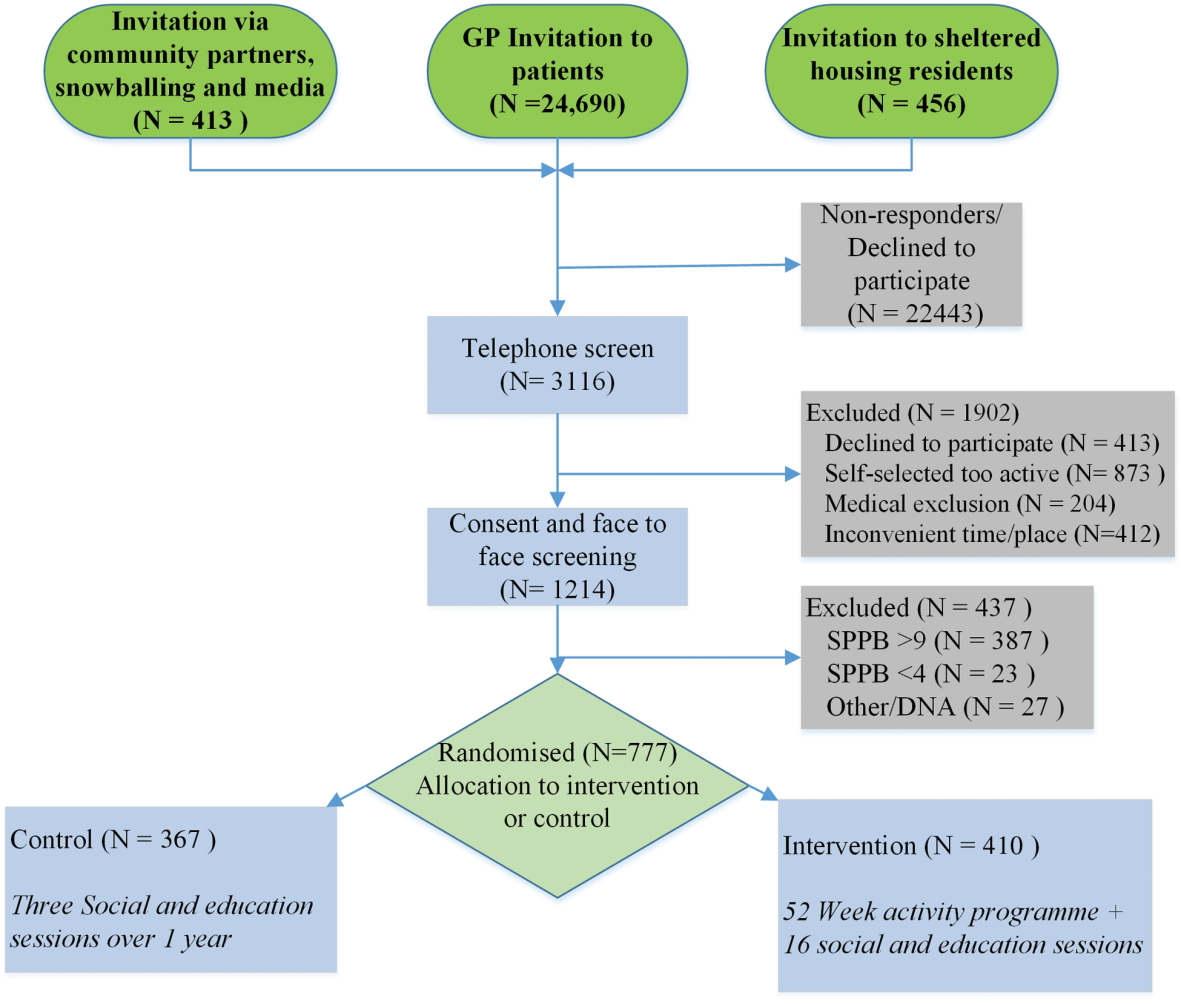
CONSORT; REACT screening, consent, enrolment, and randomization process.

Coded reply slips were issued with recruitment materials to identify the source of incoming responses. Additionally, potential participants were asked during telephone screening how they heard about the study. These data were used to generate regular reports to track the method(s) providing the greatest yield of eligible participants. The flow of participants through the study is illustrated in Figure 1.

An internal pilot study tested the effectiveness of recruitment strategies and guided any necessary adjustments made during the main trial.

### Tracking Costs

Direct recruitment costs were tracked over the 20-month recruitment period. GP invitation letters were printed, collated, and mailed by a commercial mailing company. Mailing statistics and costs were recorded on a secure website accessible to the research team. Production and distribution of invitations not issued via the mailing company, and public relations costs, were recorded. It was not possible to allocate research staff time per recruitment route, so this element is not included in the recruitment costs; however, total project staff resources were calculated.

### Statistical Analyses

Descriptive statistics were used to present the characteristics of participants and the recruitment flow from invitation to randomization.

## Results

The recruitment flow from invitation to randomization and reasons for exclusion are shown in Figure 1 and Table 1. Contrary to our estimates of contacting 9,000 (via 15–24 GP practices) potential participants to recruit 768 people over a 14-month period, we contacted 25,559 people (via 35 GP practices) over a 20-month period. Three thousand hundred and sixteen people responded to invitations and were telephone screened, 1,214 were screened face-to-face, and 777 were randomized (aged 65–98 years), slightly exceeding the recruitment goal of 768. Of the 1,902 people excluded from the study at telephone screening, 45.9% self-selected as being too active for the study, 21.7% declined to participate; for 21.6%, the time and/or place of the intervention sessions were inconvenient and 10.7% were excluded for medical reasons. Overall, the recruitment rate (invitation through to randomization) was 3.0% of those contacted compared to the predicted rate of 8.5%.

**Table 1.** Recruitment Yield—Predicted Versus Actual and Pilot Versus Main Trial

	Predicted	Actual	Pilot	Main Trial
No. of people contacted	9,000	25,559	4,467	21,092
Telephone screenings	1,980 (22.0%)	3,116 (12.2%)	838 (18.8%)	2,278 (10.8%)
Face-to-face screenings	1,580 (79.8%)	1,214 (40.0%)	228 (27.2%)	986 (43.3%)
Eligible	768 (48.6%)	777 (64.0%)	138 (60.5%)	639 (64.8%)
Overall recruitment rate	8.5%	3.0%	3.1%	3.0%
Recruitment period	14 mo	20 mo	6 mo	14 mo

### Adaptations to Recruitment During the Internal Pilot Study

The REACT internal pilot study highlighted the lower than predicted response rate from GP mailings and the limited ability to target eligible participants via GP databases. Initially, the Participant Information Sheet (PIS) was only sent out on receipt of a response/enquiry form. In the main trial, time and effort were saved by including the PIS with the initial invitation pack. In addition, the invitation letter was changed to provide a much more noticeable required participant profile in a large, prominent text box (Supplementary File 1). This reduced the response rate to the initial invitation letter by 8% between the pilot study and the main trial (18.8% vs 10.8%). However, a much higher proportion of responders in the main study progressed through telephone screening to face-to-face assessments compared to the pilot study (43.3% vs 27.2%), indicating that more suitable candidates responded.

The two main causes of exclusion were having high levels of activity/function, and inconvenience of the time or place of the intervention sessions. During the pilot study, the inclusion criteria were changed from an SPPB score of 4–8 to a score of 4–9, as those scoring 4–9 have an increased risk of future disability (21). This aligned REACT with the LIFE study inclusion criteria and increased inclusion levels at face-to-face screenings. In the main trial, the telephone screening process was adjusted to include four questions regarding ability to complete simple physical tasks such as climbing stairs and rising from a chair (Supplementary File 2). Researchers suggested to those who found all four “Easy” that they were likely to be too highly functioning for the study. Some still chose to go forward to the face-to-face assessment but the majority withdrew at that stage. The telephone script was also adapted to discuss session convenience. These steps may have helped to reduce the proportion of exclusions at face-to-face assessments from 39.5% to 35.2%.

The pilot study also underlined the importance of staggering periods of recruitment activity that enabled researchers to respond quickly. It also highlighted the need to include a prominent, well-defined description of potential participants, along with specifics of the intervention, in all communications, press releases, and interviews to avoid responses from ineligible people. For example, when a REACT interview was shown on television and accompanied by stock footage of a Tai Chi class, we received a high number of enquiries from ineligible people interested in taking up Tai Chi.

In the main trial, response rates to the initial mailed invitations were lower than in the pilot study, but as more suitable candidates responded, rates of progression from telephone screening to face-to-face screening improved considerably and eligibility at face-to-face screening was slightly improved. Despite the recruitment process taking 6 months longer than originally scheduled, the study was delayed by only 2 months. This was achieved by increasing the number of invitations issued and cancelling a planned 4-month hiatus between the recruitment phases of the internal pilot study and the main trial.

### Recruitment Strategy, Yield, and Costs

As shown in Table 2, the majority of the REACT participants (87.8%) were recruited via an invitation from their GP. The PR campaign resulted in four articles in local newspapers, one TV interview, and three radio interviews primarily in the Bath/Bristol area, leading to proportionately more recruitment via this route at this site.

**Table 2.** Total Recruitment Costs

	Bath/Bristol	Exeter/Devon	B’ham	Total	%	Cost per Recruitment Source	Cost per Recruit
Total recruited	335	268	174	777	100%		
*Participant identification*							
Invitation from GP	260	251	171	682	87.8%	£26,582.36	£38.98
PR campaign	41	1	0	42	5.4%	£726.18	£17.29
Snowballing/word of mouth	8	12	3	23	3.0%	£55.20	£2.40
Sheltered housing facilities	7	1	0	8	1.02%	£469.28	£67.04
Community partners/groups	9	3	0	12	1.5%	£52.68	£4.39
Research subject database	3	0	0	3	0.4%	£114.30	£38.10
Nonidentifiable	7	0	0	7	0.9%	£196.21	£28.03
Total cost—all recruitment sources						**£28,196.21**	
*Screening*							
Additional casual staff—telephone screening						£8,500^a^	£10.94
Venue hire per face-to-face assessment; 0.5 h/screening @ £30/h				1,214		£18,210	£23.43
Participant travel to face-to-face screenings (estimated)				1,214		£6,070	£7.81
Total						**£32,780.**	
Overall cost						**£60,976.21**	
Total cost per recruit (Total cost/777)							**£78.47**
Service Support Costs^b^						**£20,621**	**£26.54**

*Note*: B’ham = Birmingham; GP = general practitioner; PR = public relations.

^a^Estimate of the casual staff costs associated with telephone screening, ^b^Costs borne by the Clinical Research Network, not the research budget.

Total recruitment costs (including screening costs but excluding research staff time) totaled £60,976.21, yielding a per participant cost of £78.47 (see Staff section below). Costs for the different forms of recruitment are described in Table 2. GP practices provided the most productive recruitment route. They were recruited via the UK Clinical Research Network (CRN). These GPs received Service Support Costs (SSCs) from their local CRN to compensate them for the time spent on the study. SSCs varied from site to site; the mean cost per participant was £26.54. As these costs were not borne by the study, they are itemized separately in Table 2 for clarity. The mean cost of the 24,690 GP invitations mailed was 99.8p, and reply-paid postage costs for responses were £1,943, totaling £38.98 per recruited participant (*n* = 682). The printing and stationery costs for non-GP invitations were 47.9p plus 56p postage, totaling 103.9p per invitation. Invitations issued via community groups/partners cost 47.9p as there was no outgoing postage costs. The University of Bath Marketing team delivered the PR recruitment campaign at a total cost of £726.18. See Table 2 for more details.

### Staff

The staff effort budgeted across the 20-month recruitment period was 1.0 full-time equivalents (FTE) Research Associate (RA) at each of the three sites, plus a 1.0 FTE Trial Manager. When the high demands of the recruitment process became apparent, an additional short-term 0.5FTE RA post was added for the main recruitment period. The staff budget was then re-balanced, following recruitment, by reducing one RA post to 0.6FTE. Five paid casual staff and six student volunteers supported the telephone and face-to-face screening sessions.

On completion of recruitment the REACT research staff (four RAs and the Trial Manager) were asked to reflect on what they considered to be critical success factors for recruitment. These included the delivery of multiple face-to-face screening sessions giving participants date/time choices, reimbursement of travel expenses for assessments, prompt and friendly telephone contact (within 3–4 days), and building rapport and trust during telephone and face-to-face contacts. Strategies to support participants’ positive attitudes to engaging with the study were regularly discussed at research team teleconferences held fortnightly.

### Participant Characteristics

Descriptive statistics for study participants are presented in Table 4. The majority were educated beyond secondary school (417 [53.7%]), female (514 [66.2%]), overweight/obese (94 [76.5%]), and of white ethnic background (739 [95.11%]).

**Table 4.** REACT Study Baseline Characteristics

	*N*	Overall (*n* = 777)
Highest education, *n* (%)	776	
Less than secondary		64 (8.24)
Completed secondary		295 (37.97)
Some college/vocational training		206 (26.51)
College/university degree		162 (20.85)
Graduate degree, or higher		49 (6.31)
SPPB battery, mean *± SD*	777	
4 m walk score (max. 4)		3.10 ± 0.83
Balance score (max. 4)		2.93 ± 1.05
Chair-rise score (max. 4)		1.33 ± 0.85
Total score (max. 12)		7.37 ± 1.56
Self-reported Physical Function^f^, mean *± SD*	760	49.45 ± 9.35
Accelerometry, mean *± SD* MVPA^a^ (min/day)	704	5.88 ± 8.77
Self-reported PA^c^, mean ± *SD*	716	115.91 ± 57.97
BMI (kg/m^2^), mean ± *SD*	767	29.25 ± 5.71
Cognitive function^b^, mean ± *SD*	755	24.37 ± 3.66
Hand grip strength^d^ (kg), mean ± *SD*	765	25.10 ± 12.07
EQ-5D^e^, mean ± *SD*	749	0.68 ± 0.16
SF-36, mean ± *SD*	745	
Physical Component		29.85 ± 10.79
Mental Component		54.18 ± 8.49
Medical history (currently treated for), *n* (%)	764	
High blood pressure		357 (45.95)
CHD		38 (4.89)
Atrial Fibrillation		75 (9.65)
Stroke/TIA		50 (6.44)
Cancer/malignant tumour		37 (4.76)
Diabetes/high blood sugar		112 (14.41)
COPD		49 (6.31)
Mean *± SD*; physical conditions		1.46 ± 1.20
Mean*± SD*; psychological conditions		0.05 ± 0.22
Fall history (last 6 months)	760	
No. of falls, mean *± SD*		0.71 ± 1.11
Fall relating injury, *n* (%)		101 (13.00%)
Loneliness, *n (%)*	764	
Never/almost never		494 (63.6%)
Sometimes		227 (29.2%)
Often/always		43 (5.5%)

*Note*: CHD = Coronary Heart Disease; COPD = Chronic obstructive pulmonary disease; MVPA = Moderate to vigorous physical activity; SPPB = Short Physical Performance Battery; TIA = transient ischemic attack.

^a^MVPA in any bout length; ^b^Montreal Cognitive Assessment (MoCA) final score (summary of 10 cognitive tasks (46); ^c^Physical Activity Scale for the Elderly (PASE) summary score: A summary of weighted scored of 12 self-reported leisure and household activities (47); ^d^Best score of two attempts with a hand grip dynamometer; ^e^EQ-5D mean value (crosswalk score) (48); ^f^Total score of Mat-SF computer-based self-assessment of physical function (49).

### Recruitment Goals and Baseline Characteristics

The study aimed to broadly represent the diversity of deprivation and ethnicity for individuals over 65 years within the UK population. Comparisons of the REACT cohort and the over 65 years population of England and Wales are shown in Table 3 (22–24). 11.1% of REACT participants fell within Quintile 1 (most deprived) of the Index of Multiple Deprivation (IMD), compared to 14.3% of the general UK population of over 65-year olds. In Quintile 2, these figures were 20.2% and 17.6%, respectively. In terms of ethnicity, REACT under-recruited Asian participants (2.6% in over 65yrs population, 1.2% in the study) but over-recruited African/Caribbean participants (1.3% in over 65 years population, 3.0% in study). The proportion of Caucasian/white participants was slightly less than in the general population (95.1% vs 95.5%) and other/mixed ethnicities were very similar (0.8% vs 0.7%). In terms of gender, 45.6% were male compared to the 33.85% recruited into REACT. However, compared to the general population, the REACT cohort was skewed toward the older age ranges where the proportion of females increases.

**Table 3.** REACT Study Baseline Characteristics Compared to UK Population Over 65 y

	REACT (*n* = 777)	UK 65+ (*n* = 9,223,073)
**Age** (years)*, n (%)*		
65–69	95 (12.2)	2,674,161 (29.0)
70–74	191 (24.6)	2,178,672 (23.6)
75–79	190 (24.5)	1,777,547 (19.3)
80–84	160 (20.6)	1,338,005 (14.5)
85 and over	141 (18.1)	1,254,688 (13.6)
**Gender**, *n* (%)		
Female	514 (66.2)	6,617,318 (54.4)
Male	263 (33.9)	5,548,239 (45.6)
**Race/ethnicity,** *n* (%)		
Caucasian/white^a^	739 (95.1)	8,806,190 (95.5)
African/Caribbean	23 (3.0)	115,288 (1.3)
Asian	9 (1.2)	238,878 (2.6)
Other/mixed	6 (0.8)	60,872 (0.7)
**IMD** ^**b**^, *n* (%)		
Quintile 1	86 (11.1)	1,321,666 (14.3)
Quintile 2	157 (20.2)	1,618,649 (17.6)
Quintile 3	159 (20.5)	1,975,582 (21.4)
Quintile 4	156 (20.1)	2,127,763 (23.7)
Quintile 5	219 (28.2)	2,180,334 (23.6)

*Note*: IMD = Index of Multiple Deprivation. Totals and sub-totals are shown in bold.

^a^Total of all the White categories (White British, White Irish, Other White background), ^b^IMD Q1 is most deprived.

## Discussion

Recruiting older adults into active aging intervention studies is challenging. Just under half of recent UK NIHR Health Technology Assessment programs under-recruited resulting in delays, additional costs and potentially being underpowered and so unable to produce statistically meaningful results (15). In addition, under-recruitment can lead to highly selective, nonrepresentative samples consisting mainly of white and affluent participants. Non-white and less affluent groups who are more challenging to recruit engage proportionately less when studies under-recruit, so rendering results less generalizable to the wider population (25,26). In randomized controlled trials, the degree to which the final sample reflects the targeted population, details of successful recruitment strategies and learning points, as well as recruitment costs and processes all need to be clearly reported to improve recruitment success in future trials (15).

### Sample Representativeness

REACT sought to recruit a representative cohort in terms of deprivation and ethnicity. Across the IMD quintiles, we recruited 3.2% less than the general population of those most deprived (Q1), but 2.6% more than those in Quintile 2 (Table 3) (24). While not quite achieving representativeness in the most deprived group, recruiting well in the second most deprived group helped to avoid substantial skewing toward affluence.

The REACT research team had considerable experience in working with South Asian and African/Caribbean communities and were aware of the specific challenges involved. As in other studies, these issues were addressed through engaging with communities, providing funds for translators (27,28), and proportionately over-recruiting GPs in diverse areas to compensate for the typically lower response rate from ethnic groups and the most deprived. All ethnicities were recruited to population equivalent levels except Asian groups. This was likely to be due to high workloads limiting the research team’s level of community engagement, and some GP practices in diverse areas being unable to participate due to their involvement with recruiting for other studies, also keen to ensure a diverse sample.

REACT aimed to recruit older adults at risk for mobility disability. A recent expert consensus statement from the European Medicines Agency on defining frailty noted that SPPB scores of 8 or 9 indicates prefrailty, whereas SPPB scores of 7 or less classify an individual as frail (29). The change of REACT inclusion criteria to SPPB 4–9 from 4–8 ensured we targeted frail and prefrail populations and positively impacted inclusion rates at the face-to-face screenings. Inclusion criteria should be carefully considered to ensure the whole potential cohort is targeted without unwarranted restrictions (Table 4). This avoids unnecessarily prolonging the recruitment period and so increasing costs. We recruited a diverse sample in terms of functional ability; however, the cohort was skewed toward the older age ranges as compared to the general population (23), indicating that those meeting the inclusion criteria were more likely to fall within these older groups.

### Recruitment

The literature on recruitment of older adults in nonclinical trials often stresses the importance of face-to-face contact and trust building (30–32). In REACT, we found that methods such as presentations at sheltered housing facilities, relationship building, and meetings with community groups and established partners added only small numbers of participants, while requiring considerable staff resources. However, supporting existing evidence on recruitment of ethnic minorities, we found these relationship-based approaches to be valuable in supporting recruitment within diverse communities (28,32,33), although limited research staff resources affected our ability to effectively implement this approach amongst Asian groups. We would argue that in nonclinical RCTs with substantial recruitment targets, such as REACT, other recruitment methods can only ever be peripheral to large-scale approaches such as advertising, telemarketing, or a mailing program (34–38). However, we found building rapport and trust was important as potential participants passed through the screening process. A combination of large-scale and relationship-based approaches is necessary for engaging participants from low IMD and non-white backgrounds.

As reported above, targeted mailings from GPs generated the majority of REACT participants. Mass mailings such as this typically generate a 2%–6% response rate (39). REACT’s response rate was 3%. Many studies do not report mailing numbers and yields from those initially contacted. Those that do, such as STRIDE, report similar enrolment yields (3.6%) (40,41). The LIFE study could only report randomization rate from the point of telephone screening as recruitment advertising on radio, TV, and in print was widely used, meaning there was no denominator (number of people receiving invitations) to use in the calculation of initial response rates. The REACT randomization rate from the point of telephone screening was 24.9%, which compared well with the 11% achieved in LIFE (34). The evidence from the limited literature available, and the REACT recruitment process, suggests that research recruiters predicting response rates from mailing campaigns in excess of 3%–4% could be being overly optimistic, unless it is possible to select on the majority of inclusion criteria.

In future studies, more accurate targeting could substantially improve response rates and reduce costs. One means by which this might be achieved is via the United Kingdom’s electronic-Frailty Index (eFI) or similar schemes being tested in the United States (42). GPs are now required to record an e-FI for all their patients over age 65 years. The eFI is developed using the cumulative deficit model of frailty, whereby frailty is defined on the basis of the accumulation of a range of deficits, which are clinical signs (eg, tremor), symptoms (eg, breathlessness), diseases (eg, hypertension), and disabilities (43). Evidence of the eFI’s ability to capture lower extremity mobility disability is required to reveal whether its use would be beneficial in recruitment for studies targeting improved mobility.

Also, key to the success of the recruitment process were invitations and press communications that contained a prominent, well-defined description of the participants being sought, presented in layman’s terms. Once potential participants had responded to an initial invitation, it is important that the screening process was efficient, trustworthy, prompt, friendly, offered a choice of screening times, and covered travel costs (44). Regular discussions between researchers, regarding this process and feedback on it, contributed to establishing best practice and improving processes across REACT sites.

The recruitment yields in REACT were comparable with other behavioral interventions targeting similar populations (34,40). However, at £77.59 (~US$99) per randomized participant, REACT costs appear to be significantly lower than many similar studies. LIFE’s costs were $840 per randomized participant (34), Nkimbeng and colleagues reported a mean cost of $388 (38), while Ory and colleagues’ (45) mean costs varied from $103 to $939. Many of these studies were conducted in the United States where there is no equivalent to the UK’s Clinical Research Network. The Clinical Research Network recruits GP practices into its research network and compensates them for research recruitment activities via Service Support Costs (SSCs). In REACT, out of the £104.13 total cost per randomized participant, an average of £26.54 was paid in SSCs for database searching, checking of the resulting mailing list by a GP, and sending the database to the mailing house (Table 2). This very effective means of recruitment avoids the necessity of high cost techniques such as TV and radio advertising.

### Other Considerations

The REACT study is funded by the National Institute of Health Research (NIHR) Public Health Research (PHR) program. In common with other public health research funders, the NIHR PHR does not fund intervention costs. As a result, researchers working with multiple partners to deliver intervention programs are increasingly commonplace, and an excellent test of program pragmatism and sustainability. However, such partner organizations may be exposed to funding uncertainty, staff changes and organizational challenges. No formal contractual relationship existed with partners whose involvement was voluntary and unfunded. During REACT the main Birmingham delivery partner reduced their commitment from delivering nine to two intervention groups due to funding issues. Three new partners were ultimately recruited across the wider Birmingham area to deliver an additional five groups, but the final two groups were moved to the Bath/Bristol site to avoid delaying the study further. Fortunately, it was possible to adjust staff resources to cope with this additional workload.

This article focuses on the tribulations of recruitment of participants to trials. Lessons learnt should be generalized with caution when designing recruitment strategies for real life community programs where timescales, motives, and support for participation might differ from those of randomized controlled trials.

## Conclusion

The REACT RCT successfully recruited to target demonstrating that it is feasible to recruit at-risk, community-dwelling older adults to a large public health intervention study, targeting mobility disability. An internal pilot study was important in fine-tuning the recruitment and screening processes.

Steps taken to improve recruitment yields here may be useful in other studies seeking to recruit older, frail or prefrail populations. We found that GP mailings were a reliable and adaptable form of recruitment that could be increased or reduced to meet targets and match available staff resources. However, choice of recruitment pathways should take into account relative effectiveness, cost and resource requirement, while the additional benefits of methods, such as face-to-face engagement with some non-white and less affluent older adult populations, need to be considered. REACT response rates were lower that initially predicted and as a result the recruitment period was extended highlighting that when planning large trials, response rates and recruitment timescales need to be realistic and calculated based on data from similar studies. To facilitate this all trials should be required to provide recruitment data on completion of their recruitment phase.

## Funding

This work was supported by the National Institute for Health Research, Public Health Research Programme (13/164/51).

## Supplementary Material

glaa051_suppl_Supplementary_letter_V1

glaa051_suppl_Supplementary_letter_V2
