# Supplementary material for: The Tribulations of Trials: Lessons Learnt Recruiting 777 Older Adults Into REtirement in ACTion (REACT), a Trial of a Community, Group-Based Active Aging Intervention Targeting Mobility Disability
Source: J Gerontol A Biol Sci Med Sci. 2020 Mar 9;75(12):2387–95. doi: 10.1093/gerona/glaa051 (PMC7662171; doi:10.1093/gerona/glaa051)
Supplement: glaa051_suppl_Supplementary_letter_V1 [file glaa051_suppl_supplementary_letter_v1.docx]

GP Letterhead **Letter from GP to potential participants V1**

<<Date>>

<<Patient name>>

<<Address>>

**Can we help older people maintain their mobility and live independently for longer?**

**Help us to find out…**

Dear <<title>> <<surname>>

I am writing to invite you to take part in a research project called REACT (Retirement in ACTion). As people get older, losing the ability to do everyday activities makes it more difficult to live independently and can seriously affect people’s quality of life. However there is a lot of research to show that it is possible to stop this decline in function, even reverse it, by keeping people active. The REACT programme is designed to support people to gently become more active in a supportive and sociable setting. So if walking, climbing stairs and getting up from a chair are starting to get more difficult for you, you might be interested in taking part in our research, to see if REACT works.

If you take part in the research, you will be asked to do one of two things.

- Either you will be asked to attend physical activity and social sessions, with a small group of other older people, at a local community centre
- Or will be given an information pack including advice on keeping healthy as you get older, details of appropriate local activities and you will be invited to three social and health education sessions.

Whichever of these two groups you are in, a researcher will also ask you to

a) provide some information about yourself (the researcher will go through a questionnaire with you)

b) take part in some simple physical function tests (a 4 metre walk, going from sitting in a chair to standing, and a balance test) and

c) ask you to wear an activity monitor around your wrist for a week.

REACT is being delivered by the Universities of Bath, Birmingham and Exeter, and almost 800 people will be participating. There is no obligation to take part in the research. However, please give it some careful thought as we believe taking part offers several benefits for you.

At this stage, we just want to know if you are interested in getting more information. If you are, please return the attached reply slip in the postage paid envelope provided (this is free - no stamp is needed) or contact *(Local research assistant on ???).* The research team will then send you more information to help you decide about taking part and will then contact you by phone a few weeks later to see if you are interested. In the meantime please feel free to discuss this with me, other healthcare staff, or your friends and family**.**

Yours sincerely

Dr <<GP name>>
